# Estimation of Bait Uptake by Badgers, Using Non-invasive Methods, in the Perspective of Oral Vaccination Against Bovine Tuberculosis in a French Infected Area

**DOI:** 10.3389/fvets.2022.787932

**Published:** 2022-03-09

**Authors:** Ariane Payne, Sandrine Ruette, Mickaël Jacquier, Céline Richomme, Sandrine Lesellier, Sonya Middleton, Jeanne Duhayer, Sophie Rossi

**Affiliations:** ^1^Wildlife Disease Unit, French Office for Biodiversity, Orléans, France; ^2^Groupement de Défense Sanitaire de Côte d'Or, Breteniere, France; ^3^French Office for Biodiversity, Predators and Alien Species Unit, Birieux, France; ^4^Claude Bernard Lyon 1 University, CNRS UMR5558, LBBE, Villeurbanne, France; ^5^ANSES, Nancy Laboratory for Rabies and Wildlife, Malzéville, France; ^6^Animal and Plant Health Agency, Woodham Lane, United Kingdom

**Keywords:** badger *(Meles meles)*, bovine tuberculosis, bait deployment, hair trapping, biomarker, oral vaccination

## Abstract

Although France is officially declared free of bovine tuberculosis (TB), *Mycobacterium bovis* infection is still observed in several regions in cattle and wildlife, including badgers (*Meles meles*). In this context, vaccinating badgers should be considered as a promising strategy for the reduction in *M. bovis* transmission between badgers and other species, and cattle in particular. An oral vaccine consisting of live Bacille Calmette–Guérin (BCG) contained in bait is currently under assessment for badgers, for which testing bait deployment in the field and assessing bait uptake by badgers are required. This study aimed to evaluate the bait uptake by badgers and determine the main factors influencing uptake in a TB-infected area in Burgundy, north-eastern France. The baits were delivered at 15 different setts located in the vicinity of 13 pastures within a TB-infected area, which has been subject to intense badger culling over the last decade. Pre-baits followed by baits containing a biomarker (Rhodamine B; no BCG vaccine) were delivered down sett entrances in the spring (8 days of pre-baiting and 4 days of baiting) and summer (2 days of pre-baiting and 2 days of baiting) of 2018. The consumption of the marked baits was assessed by detecting fluorescence, produced by Rhodamine B, in hair collected in hair traps positioned at the setts and on the margins of the targeted pastures. Collected hairs were also genotyped to differentiate individuals using 24 microsatellites markers and one sex marker. Bait uptake was estimated as the proportion of badgers consuming baits marked by the biomarker over all the sampled animals (individual level), per badger social group, and per targeted pasture. We found a bait uptake of 52.4% (43 marked individuals of 82 genetically identified) at the individual level and a mean of 48.9 and 50.6% at the social group and pasture levels, respectively. The bait uptake was positively associated with the presence of cubs (social group level) and negatively influenced by the intensity of previous trapping (social group and pasture levels). This study is the first conducted in France on bait deployment in a badger population of intermediate density after several years of intensive culling. The results are expected to provide valuable information toward a realistic deployment of oral vaccine baits to control TB in badger populations.

## Introduction

The ability of some wild hosts to maintain or transmit pathogens to livestock is often a barrier to control and eradication of many major veterinary diseases, because influencing the wild hosts' behavior, ecology, and susceptibility to the pathogen is very difficult. In wildlife, the feasibility of surveillance and control measures are constrained by a limited access to animals and any intervention in natural ecosystems might be controversial (1, 2). Depending on the pathogen transmission dynamics, the host species' specificities, and the available diagnostic and control tools, mitigation measures may include reduction of naïve or infected hosts' density, a reduction in contact rates between wild hosts and/or at the wildlife-livestock interface by lethal control, vaccination, and/or barriers and biosecurity measures (2). The oral vaccination of wildlife has been an effective strategy to control rabies in red foxes (*Vulpes vulpes*) and classical swine fever (CSF) in wild boar (*Sus scrofa*) (3–6). However, developing effective and safe oral vaccines for wildlife is very challenging, because the vaccine bait must satisfy a number of requirements: be palatable to the host, deliver an effective vaccine dose with a suitable shelf life, be compatible with a safe and efficient release of the vaccine to the oral mucosa, be affordable, be user-friendly, be stable, and be safe for the environment (7).

*Mycobacterium bovis* is a multi-host pathogen infecting a wide range of mammals, including the European badger (*Meles meles*). Although France is officially bovine tuberculosis (TB)–free since 2001 (i.e., herd TB prevalence at less than 0.1% at a national level), some cases have still been occurring locally in both cattle and wildlife. The number of outbreaks has increased since 2004, especially in the Southwest of the country. Currently, localized outbreaks continue to be reported in the Northeast (8). The French *M. bovis* host community comprises cattle, badger, wild boar, deer (*Cervus elaphus* and *Capreolus caprelolus*), and foxes. Badgers have been identified as spillover hosts, able to transmit the infection to cattle, due to their capacity to excrete *M. bovis*, the interactions existing between the two species, and the occurrence of identical strains locally in both species (9–11). In north-eastern France, *M. bovis* infection persists locally at the farm level in cattle and badgers despite 9 years of badger culling and management measures implemented in cattle. In the context of likely *M. bovis* transmission from infected badgers to cattle, vaccinating badgers is an attractive tool to limit the transmission of *M. bovis* within the host community. Delivering a vaccine by parenteral route requires live-trapping and injection of individuals and is thus labor-intensive and time-consuming. As for other wildlife vaccines (e.g., rabies and CSF), the oral delivery through bait would be an attractive method to administer the vaccine to badgers relatively easily.

BCG (Bacille Calmette–Guérin) is currently the main available vaccine against TB and one of the most widely delivered vaccines worldwide. An injectable form of BCG was licensed in 2010 for badgers in the UK where the species is considered to be a maintenance host, which hampers the eradication of TB. Culling (targeted or wide-scale) has been implemented with contrasting effects on cattle incidence and has raised ethical issues (12). Models simulating different control strategies in badgers (culling at various scales and/or vaccination, with or without preliminary testing) in the endemic areas of the UK and the Republic of Ireland showed that vaccination, if combined with culling to ensure a low population density, is an effective tool to reduce prevalence in badgers and incidence in cattle (13–15).

This evidence justified international investments in the development of a more sustainable control strategy against TB such as vaccination with BCG. Evidence for a protective efficacy by BCG in the field in badgers was demonstrated after injection (16) and oral delivery (17), including in non-vaccinated cubs of vaccinated groups (18). In captive studies, BCG, administrated either by the parenteral or oral route, reduced the severity of the experimentally induced disease and the excretion of bacilli (19–21).

A number of different palatable bait products have been evaluated in the UK, as potential components of a bait-vaccine product for the oral vaccination of badgers against bovine TB (22–24) and a lead oral vaccine delivery product was identified, comprising a lipid vaccine carrier embedded in a highly palatable peanut-based custom bait (23). An optimal delivery strategy to maximize the bait uptake by badgers, ensuring sufficient vaccine coverage within the target population while limiting the cost, was important in this selection: an uptake by badgers (based on biomarking methods) ranging from 75 to 98% with a high variability among social groups was reported (24–26).

The present study aimed to evaluate bait uptake by badgers within an infected area in north-eastern France where TB has persisted at low levels (less than 10 new infected herds per year and less than 5% prevalence in wildlife since 2017, see Section Study Site) and where badgers have been culled for 9 years. Here, we focused on the badger–cattle interface by identifying badger social groups living in the vicinity of in-use pastures. The aim of this study was to determine the proportion of badgers that had consumed the baits at three levels: (1) individual, (2) social group, and (3) the pasture level. We aimed to determine how the bait uptake varied in relation to the social group's characteristics (group size, presence of cubs, and use of an outlier sett) and deployment strategies (number of baits deployed, number and season of deliveries, and selection of the setts in relation to the corresponding pasture). Data generated by this study will help to determine the optimal deployment strategy adapted to local epidemiological and ecological contexts in France.

## Materials and Methods

### Study Site

**Figure 1:**
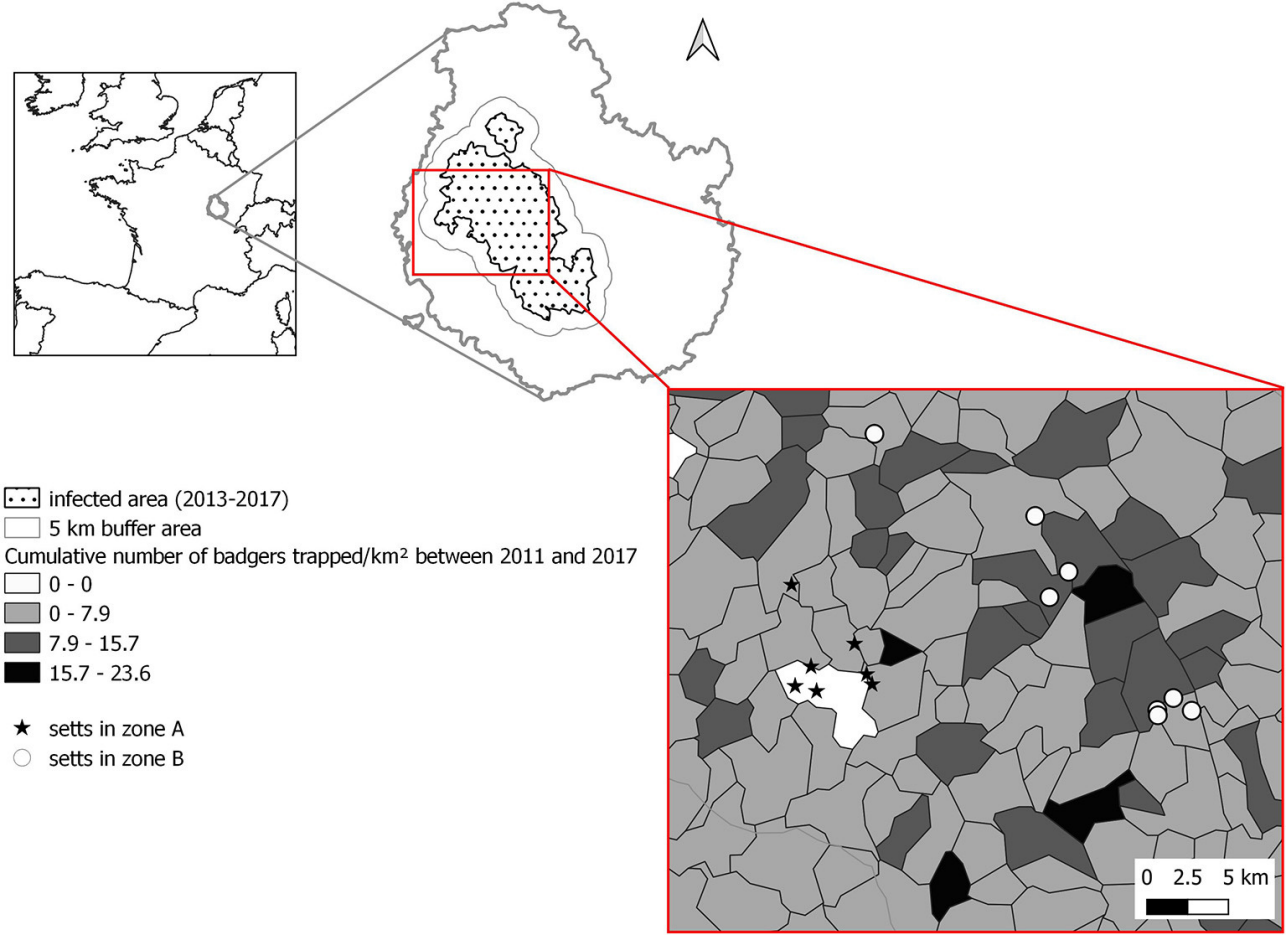
Location of the setts selected within the study zone.

The study site was located in the French administrative division of Côte d'Or in the Burgundy region of the north-east of France (Figure 1). In this area, TB has been circulating in the host community including cattle and wild species, i.e., badger, wild boar, red deer, and, more sporadically, fox (9, 11). The bait deployment area of this study covered 200 km^2^ of the whole infected area of Côte d'Or (3,000 km^2^) and was composed of mixed woodland and agricultural land (mainly pastures). In the study area, TB incidence has been decreasing over the past 9 years following control measures implemented in livestock and wildlife (intensive culling of both badgers and infected cattle), but eradication has not been achieved because of local persistent hot-spots: The number of infected farms dropped from 45 in 2010 to 3 in 2018 (8), whereas prevalence measured by PCR was 8.1% in 2013–2014 and 4.2% in 2016–2017, 3.1% in 2011–2012 and 2.4% in 2016–2017 in badgers and wild boar, respectively (11).

Since 2010, badger culling has been implemented as part of the management measures to control the spread of TB to and from cattle. This control measure has been implemented within the infected area encompassing 2-km radius around pastures with cattle outbreaks or around trapping locations of infected badgers detected in the previous 4 years. A “buffer area” was also defined in a 5-km radius around the infected area (Figure 1), where badgers have been trapped with a surveillance objective only. Between 2009 and 2017, an average of 0.97 ± 0.50 badger/km^2^ were culled within the risk area each year and with an average of 0.52 ± 0.61 badger/km^2^ culled each year in the buffer zone (Supplementary Material 1). The intensity of culling was, however, heterogenous among municipalities [source: local veterinary services and (27)] (Figure 1). At the north western boundary between the infected and the buffer area (called zone “A” thereafter), badgers' density [estimated by using a combination of distance sampling, camera trapping, and hair trapping for genetic identification, see (28) for details] was estimated in 2017 at 2.57 adults/km^2^, whereas, in the core of the infected area (called zone “B” thereafter), the badger density was evaluated in 2017, at 3.79 adults/km^2^ (28). In both zones, the proportion of occupied setts decreased between 2012 and 2017 from 0.73 to 0.60 in zone A and from 0.79 to 0.55 in zone B (27, 28). Regarding the density of adult badgers, it decreased in zone A by 26%, whereas it was multiplied by 1.5 in zone B (most likely because the peak of culling intensity was reached in 2012 and then decreased, as shown in Supplementary Material 1) (27, 28).

### Sett Selection

To target the badger–cattle interface, we selected 15 badger setts in the vicinity of 13 pastures (mean area: 16.7 ± 10.7 Ha) used by cattle, of which six belonged to farms that had at least one breakdown in the last 9 years. Seven setts were selected within zone A and eight within zone B (Figure 1). The setts were identified during a survey ranging up to 300 m from the pasture fence depending on accessibility. The setts were located at an average of 45 m from the pasture boundary (range 0 to 185m).

Badger setts usually comprise one main sett that serves as the primary year-round residence and other smaller (outlier) setts that tend to be occupied less frequently (29). All outlier setts showing signs of badger activity and falling within radius of 300 m of the recruited main setts (26) were also included in the study. Among the 15 selected main setts, six had an outlier sett identified this way. All individuals identified either at the main sett and/or at the outlier sett when present were considered to belong to the same social group.

Trapping activity (for culling or surveillance purposes) using stopped restraints was ongoing during our study on seven setts (three in zone A and four in zone B) but not during bait deployment.

### Bait Preparation and Deployment

**Figure 2:**
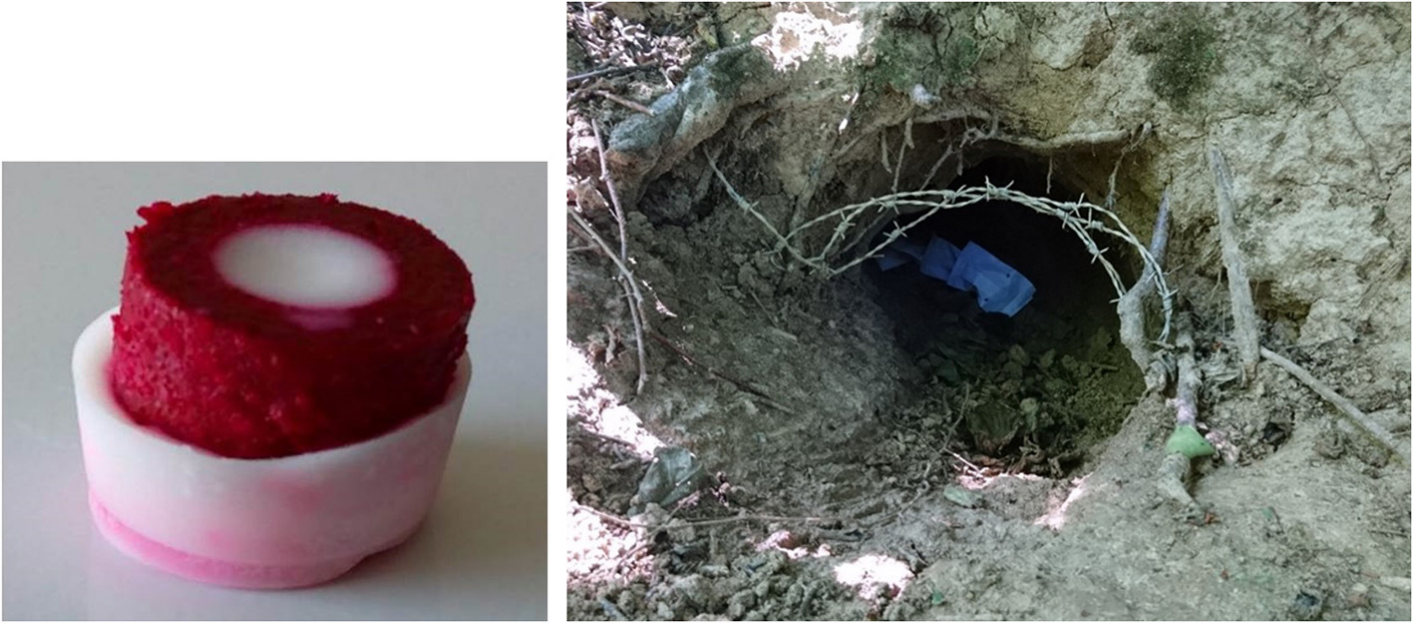
Bait produced by Connovation Ltd. (Manukau, New Zealand) used in the study. On the left picture (by Rémi Schmitd), the palatable part (or PT made of peanut butter, cereal, and sugar) appears in red as it also contains Rhodamine B. The white part corresponds to the hardened peanut oil (HPO) where the vaccine, when present, may be inserted in the center. Right picture (by Matthieu Colombe): hair trap and baits deployed in the hole of a badger sett. These baits were previously packed within an YPBFERAL^®^ paper bag.

The candidate bait developed by the Animal and Plant Health Agency (APHA, Weybridge, UK) for vaccinating badgers against TB (23) was used (Figure 2). The palatable part of the bait (Paste bait or PT) is based on a proprietary recipe and was developed by Pest-Tech^®^ Ltd., New Zealand, and produced by Connovation Ltd. (Manukau, New Zealand). The main components are peanut butter, cereal, and sugar. PT has a hollow cylindrical shape (hoop), filled in the center with hardened peanut oil (HPO) where the vaccine may be inserted [with a dose conferring protection in badgers, i.e., 10^8^ CFU (21)]; in this study, none of the baits contained any vaccine. The total weight of the PT-HPO bait is 15 g (23, 24). A biomarker, Rhodamine B (RhB; Sigma-Aldrich, Dorset, UK) was mixed with the dry ingredients of the PT bait during manufacture of the bulk material at Connovation, for a final a concentration of 100 mg per bait. The final preparation of the baits was carried out at APHA. This biomarker has previously been employed to successfully measure bait uptake in badgers (24, 25, 30, 31). The RhB is safe, can be detected rapidly after ingestion (1 day post-baiting) and for several weeks in hair (30, 32), and can be mixed with the PT without any loss of palatability (33).

To reduce neophobia and increase bait uptake, pre-baits made of PT only, without RhB, were deployed, before delivering the PT-HPO baits (24, 25, 34). We deployed the baits in the spring for 15 setts and in the summer for a subset of 10 setts (five in each zone A and B) to be able to determine if two deliveries would increase bait uptake. Spring and summer were chosen as seasons when the resource availability is lowest for badgers and when weaned cubs emerge to start foraging (25, 26). The duration of pre-baiting, baiting, and the number of baits delivered per sett were based on protocols developed by APHA [(25, 26, 31) and Robertson, pers.com].

#### Spring Deployment

**Figure 3:**
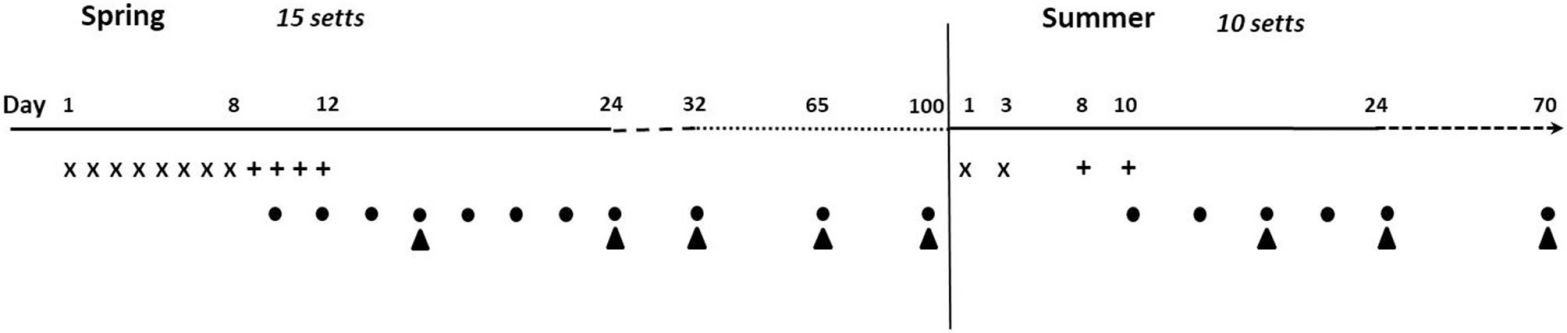
Schedule for bait deployment and hair collection in spring (March to April 2018) and summer (July to August 2018). **X**, pre-baits deployment; **+**, baits deployment; •, hair collection near setts; ▴, hair collection on pastures.

In each zone, pre-baits were delivered daily for 8 days. On the ninth day, pre-baits were replaced by the PT-HPO baits, which were then deployed every day until day 12 (Figure 3). Because of logistical constraints, zone B was baited first, followed by zone A. Pre-baits and baits were delivered directly inside sett entrances to limit access to non-target species (mainly red fox and birds), following a protocol developed in the UK (35). The number of active holes per sett was highly variable (range: 2–30). As a consequence, the sett size was scored depending on the number of active holes (1 = less than 5, 2 = between 5 and 10, and 3 = more than 10 active holes) and assigned a number of pre-baits and baits per day for each score (6/14/20 pre-baits and 15/21/30 baits, respectively). Active holes of the outlier setts were added to the score of the whole sett cluster and were also baited. Two to four pre-baits and baits were delivered per active hole. Uneaten baits were left on site. A total of 2,000 pre-baits and 1,212 baits were deployed in spring. Baits were not packaged.

#### Summer Deployment

In summer, the protocol was modified to compensate for the sharp decline in the observed level of badger activity following the spring bait deployment at several setts (mainly in zone B). In addition, one sett used in the spring in zone B was not used in the summer because of a lack of activity. Assuming that human disturbance during bait delivery was responsible for this decline, the number of deployment days in summer was limited: pre-baits were delivered on days 1 and 3 and baits on days 8 and 10 (Figure 3). Given that badgers had already been familiarized with the baits in spring, the pre-baiting period was reduced for the summer deployment (Figure 3). The number of baits delivered per day was increased (sett score = 1:16/2:28/3:40 baits) to compensate for having halved the number of deployment days. A total of 484 pre-baits and 536 baits were deployed at 10 setts between July 9 and July 19. As for the spring deployment, pre-baits and baits were thrown inside the active holes. Contrary to spring when baits were not packaged, each summer bait was distributed in an YPBFERAL^®^ paper bag (75 × 150 mm, perforated with a single hole punch and folded over two to three times), to reduce the bait disintegration at higher temperatures. Given that the badgers had already been familiarized to the baits in spring and UK available data evidence that the bags did not deter badgers from consuming baits (22), it was considered that the bags would not compromise attractiveness of baits to in summer.

### Camera Trapping

Camera traps were used to monitor badger activity and to check for the presence of cubs at the baited setts. Forty infrared motion triggered camera traps (25 StealthCam^®^ G42NG, 10 Cuddeback^®^ Attack IR, 3 Bushnell^®^ Trophy Cam and 2 ScoutGuard^®^ 560k) were used. They were programmed to work continuously from March 8 to August 8, 2018, to cover 11 days before the first bait deployment and 20 days after the second with a passive infra-red (PIR) motion sensor delay set to 5 s and to record 20 or 30 s of video footage. Depending on the sett size, activity, configuration, and bait deployment strategy, one to four camera traps were set per sett. They were fastened to trees at a height ranging from 1 to 1.60 m with a downward pointing angle to target holes where baits were delivered. Batteries and SD cards were checked at the end of each phase of the bait deployment protocol.

A visit was defined as the observation of one or more individuals within a 15-min recorded time interval. For each visit, the maximum number of individuals and the presence or absence of cubs was recorded.

Badger activity was estimated by dividing the number of badgers' visits by the number of camera-trap days during the spring and summer deployments and between the two deployments. The badgers' activities between zones A and B and between social groups with cubs vs. no cub were compared using a Wilcoxon test.

Camera traps were stolen and not replaced on one sett before the spring deployment. As a consequence, badger activity was estimated on 14 setts during the spring bait deliveries and on 13 setts in summer (as mentioned before, one sett was excluded from the deployment in summer because of reduced activity).

### Hair Collection

The bait uptake was quantified using a non-invasive method, on the basis of hair collection in hair traps without trapping animals, where hairs were examined for a biomarker (RhB) and analyzed genetically to identify individuals.

Badger hair samples were collected from the 15 selected setts and their outliers, using hair traps (36). The traps were made of two interwoven strands of barbed wire (thickness of 1.7 mm, with barbs spread every 10 cm) suspended approximately 18 cm above ground level across sett holes and visible badger runs, with a length ranging from 30 cm to 1 m (Figure 2), using natural elements such as trees when possible and wooden stakes if necessary. They were set up 1 or 2 months before bait deployment to limit neophobic behavior, and all pre-study samples were removed. Depending on the sett size, the number of active holes and active runs, between 5 and 18 hair traps, was installed per sett (mean = 11 ± 4 SD), with a total of 166 traps.

Hair traps were also placed above well-frequented badger runs, leading to the 13 pastures close to the selected setts with the aim of investigating whether badgers visiting the pastures had consumed the baits. Pastures were used by cattle during the study, but hair traps were laid in such ways to prevent interactions between cattle and traps. Depending on the size of the pasture and the number of badgers runs detected, we set between 5 and 36 hair traps per pasture with an average of 17 (± 10 SD) and a total of 221 traps for all the pastures. Each hair trap was labeled and geolocalized.

In spring, hair samples were collected over a period of 2 weeks, every 2 days from the setts, and every week on pastures from the first day following the bait deployment. Hairs were then collected once on the third week and then once a month until the summer deployment. In summer, hairs were sampled five times between day 3 and day 25 and finally 1.5 to 2 months later depending on the sett and pasture. Each sett (including the outlier setts and the five setts that had not been baited in summer) and each pasture were sampled the same number of times (Figure 3).

Hairs attached to one single barb were considered as corresponding to one individual. This assumption was based on previous study where 5.5% (41/741) of the hair collected on one single barb were mixed (e.g., genetic pool of different individuals) (37). When four or more hairs were collected per barb, the sample was roughly divided into two for RhB detection and for genetic typing, respectively. This was to limit contamination of the genetic material when handling the samples. However, when the number of hairs was three or less, the same sample was reused for RhB followed by genetic typing.

As badger trapping for TB control was ongoing after the bait deployment at seven out of the 15 setts included in the study, trappers were asked to take hair samples from any individuals trapped within the 2-km radius of the baited setts. The GPS coordinates, the sex, and the age (young/adult) of the trapped badgers were also recorded. All hair samples were stored at room temperature before analysis.

### Genotyping of Badger Hair Samples

Genotyping was performed by the Antagene laboratory (La Tour-de-Salvagny, France). See (37) for further details on the sample preparation, DNA extraction, and PCR reaction. For each DNA sample, 24 microsatellite markers (detailed in Supplementary Material 2) and one marker for sex identification (SRY) were amplified by two multiplex PCRs and analyzed with an automated sequencer in two migrations. The electropherograms were analyzed using GENEMAPPER 4.1 (Thermo Fisher Scientific) and analyzed independently by two analysts to determine the allele sizes for each marker of each individual.

### Individual Identification

To identify individual badgers, we used the genotypes obtained from collected hair samples. At least eight successfully genotyped markers (out of 24 amplified) were used to derive consensus genotypes. Genotypes were regrouped using the GIMLET v1.3.3 software (38) following the method described in (35). We then estimated the probability of identifying siblings PI_sib_ (39) from all obtained consensus genotypes. This probability allowed us to determine the confidence for distinguishing individuals.

### Rhodamine B Detection

To assess bait uptake, hairs collected from traps were examined under a fluorescence microscope (Olympus BX41) at ×4 magnification with a RhB filter in a dark room. If the sample contained less than four hairs, then they were all examined. In case of more than four hairs, a subsample of four was selected if possible of different hair types (i.e., guard hairs and fine hairs). Each hair was examined from the bulb to the extremity to look for RhB staining. If the RhB was not consistently detected among the hairs in the sample (i.e., there were RhB-positive and RhB-negative hairs within one single sample), then more (with a maximum of 10) were examined. Hairs collected on badgers coming from other zones where no marked baits with RhB had been delivered were used as negative controls. A sample was considered positive for RhB if at least one hair belonging to the sample fluoresced in the root and/or along the hair shaft (32) (see Supplementary Material 3). The examination was carried out independently of the genetic typing results, in a blinded manner. An individual was considered positive for RhB and, therefore, having consumed one or more baits when at least one of its hair samples was positive for RhB.

### Statistical Analysis and Definition of the Variables

From the number of individual badgers positive to RhB and the total number of individuals identified genetically, we computed the bait uptake (i.e., the proportion of positive badgers) for the whole study zone, and the mean proportion of positive badgers per sett and per targeted pastures.

- At the individual level, we investigated which factors could influence the individual bait consumption using a generalized linear mixed model (GLMM). The response variable was the RhB result for that individual sample (binary, positive = 1, negative = 0), indicating whether the badger had consumed baits (1) or not (0). The explanatory variables included the *sex*, the number of hair samples collected for each individual (*samples*), the number of individuals hair trapped at each sett as a proxy for *group size* (although trapping efficiency is unlikely to be 100%), the presence of the individual at the outlier sett (*caught at outlier sett*) when present (i.e., if at least one hair sample belonging to one individual was collected at the outlier sett), the number of *seasons* the sett was baited (one in spring or two in spring and summer), and the *zone* (A or B) (Table 1). The *social group* of the individual was included as a random effect to take into account the likely dependence within one group. All combinations of explanatory variables were evaluated and ranked using Akaike's information criterion (adjusted for small sample sizes; AICc). We selected a top model set from models having a ΔAICc less than 4 (from the top model). Average model parameters were calculated using this top model set and variables classed as having a significant effect if the 95% CI of the coefficients did not span zero (40). All analyses were performed by using lme4 and MuMIn packages in R 3.4.2 software (41).
- At the social group and pasture scale, we used a factor analysis with mixed data (FAMD) to explore the similarities among the 15 social groups and the 13 targeted pastures with a particular interest toward variables that may be correlated with the proportion of RhB-positive badgers per sett (*Prop_pos_sett*) and the number of RhB-positive badgers per pasture (*Nb_pos_pasture*). We then assessed correlations using univariate analysis, i.e., Wilcoxon test for qualitative variables and Pearson correlation tests for quantitative ones. The studied variables are displayed in Table 1.

**Table 1:** Definition and type of the variables used to investigate the bait uptake by badgers at the individual (in a generalized linear mixed model), social group, and pasture levels (in factor analysis with mixed data).

**Variables**	**Definition**	**Type**	**Individual level**	**Social group level**	**Pasture level**
**Individual characteristics**					
Samples	Number of samples collected per individual, standardized by the length of the collection period (Min: 0.5, Max: 9 samples/100 days)	Quantitative	X		
Sex	Female/Male determined genetically	Qualitative	X		
Caught at outlier sett	Individual caught at the outlier sett when present (Yes/No)	Qualitative	X		
**Social group characteristics**					
Social group	Individuals are assigned to one social group when caught at the same sett	Qualitative/random effect	X		
*Prop_pos_sett*	Number of RhB-positive badgers/group size for each social group	Qualitative		X	
Outlier	Presence of active (and baited) outlier sett (Yes/No)	Qualitative		X	
*Group_size*	Number of individuals identified genetically on a given sett	Quantitative	X	X	
Cubs	Cubs detected by video surveillance on a given sett (Yes/No)	Qualitative		X	
Spring_activity^*^	Number of badgers visits/camera-trap day during the bait deployment in spring	Quantitative		X	
Trapping	Ongoing trapping activity (Yes/No)	Qualitative		X	
**Pasture characteristics**					
*Nb_pos_pasture*	Number of RhB-positive badgers identified on a given pasture	Quantitative			X
Area	Area of the pasture	Quantitative			X
*Dist_sett.pasture*	Shortest distance between the main sett and the pasture border. Two classes: Dist 1 = 0 m and Dist2 > 0 m	Qualitative			X
Prospection_rate	Prospected area computed from GPS tracks data/300-m buffer area around the pasture computed by QGIS.	Quantitative			X
*Nb_badgers*	Number of individuals identified genetically on a given pasture	Quantitative			X
**Area characteristics**					
Zone	Zone A/Zone B	Qualitative	X	X	X
*Prev_trapping*	Number of badgers culled/km^2^ between 2011 and 2017 in the municipality where the sett was located	Quantitative		X	X
**Delivery strategy**					
Seasons	Bait delivery in Spring (del1)/in Spring and Summer (del2)	Qualitative	X	X	X
*Nb_baits*	Total number of baits deployed per social group or per pasture (equivalent to the number deployed per social group except when two social groups surrounded a single pasture)/group size or number of badgers identified on the pasture	Quantitative	^**^	X	X

* *Camera traps were stolen at one sett. We used the averaged value of the spring activity recorded on other setts to replace this missing data. The summer activity was not included in the analysis as one sett showed a complete loss of activity before the summer deployment and was excluded from the study for this period. As a result, the analysis could not be performed using this variable*.

** *This variable did not allow to correctly fit the model because of singularity and was not included in the model selection*.

## Results

### Camera Trapping

Cubs were detected at 10 out of the 15 setts. The maximum number of individuals observed in one visit ranged from 1 to 6 (mean = 3.0 ± 1.7 SD) (Table 2).

**Table 2:** Ecological and baits delivery characteristics for each social group.

**Social group ID**	**Zone**	**Number of delivery**	**Fed outlier sett**	**Cubs**	**Badger activity^**a**^ during the spring deployment**	**Badger activity^**a**^ during the summer deployment**	**Max no. of badgers observed on video**	**Group size (from genetic typing)**	**No. of badgers RhB positive**	**No. of baits available per badger^**b**^**
1	B	2	No	Yes	0.04	0	4	4	3	29
2		1	No	No	0	NA^c^	1	1	0	84
3		2	No	Yes	0	0.06	2	2	2	58
4		2	Yes	Yes	0.07		2	8	3	17.5
5		1	Yes	No	0	0.01	1	2	0	30
6		2	No	Yes	0	0.31	4	6	1	19.3
17		1	No	No	0.03	0	2	2	1	30
18		2	No	No	0	0	2	0	0	NA
10	A	2	Yes	Yes	0.33	0	6	9	4	15.6
12		2	No	Yes	0.78	0.08	5	6	5	27.3
13		2	Yes	Yes	0.78	0.28	3	6	4	19.3
14		1	Yes	Yes	0.02	0.03	3	8	3	15
15		2	No	Yes	NA^d^	NA^d^	NA^d^	8	8	20.5
16		2	Yes	Yes	0.21	0.05	6	14	8	10
19		1	No	No	0.06	0.05	1	6	1	20
Mean ± SD					0.17 ± 0.28	0.09 ± 0.12	3.0 ± 1.7	5.5 ± 3.7	2.9 ± 2.6	28.2 ± 19.7

*The badger activity and maximum number of badgers were derived from video surveillance, whereas the group size was drawn from the genetic typing*.

a *Number of visits per day*.

b *Number total of baits delivered on the sett per group size*.

c *Not monitored during summer*.

d *Camera-traps stolen*.

**Figure 4:**
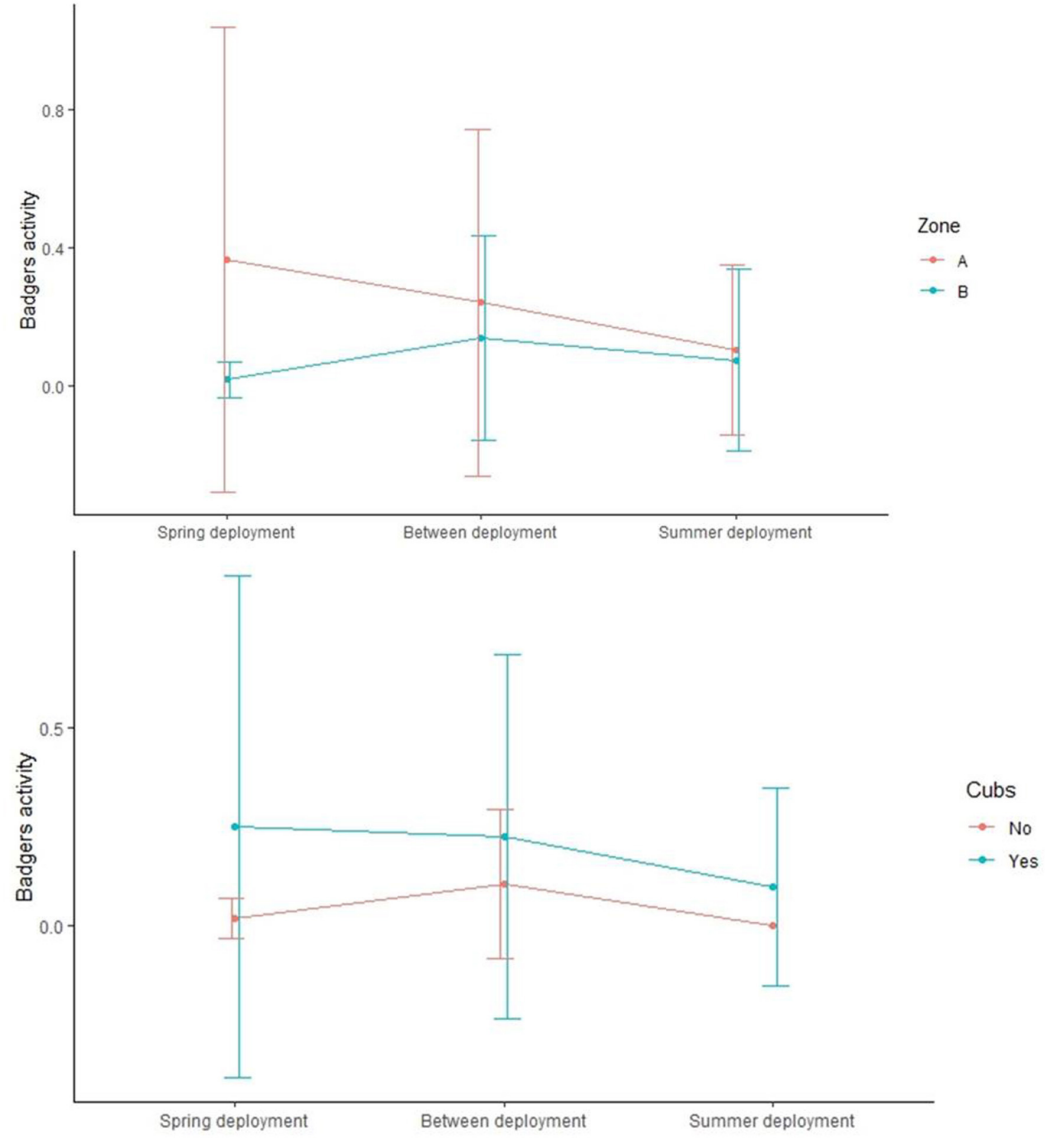
Mean (± 95% CI) badger activity at social groups in zone A compared to zone B (top panel) and where cubs were detected or not by video surveillance (bottom panel).

The level of badger activity (measured as number of visits per day) at monitored setts was on average 0.17 ± 0.28 SD, 0.09 ± 0.12 SD, and 0.18 ± 0.20 SD during the spring, summer, and in between deployments, respectively. The activity was significantly higher in zone A than in zone B (Wilcox.test, *p* = 0.006) and in setts where cubs were present (Wilcox.test, *p* = 0.05) (Table 2 and Figure 4).

### Hair Samples

A total of 646 hair samples were collected between March 28, 2018, and October 5, 2018. Of these, 397 were collected on setts, with a range of 0 to 63 samples per sett (mean = 26.5) and 242 hair samples on pastures (mean per pasture = 16.1, range: 0–49). Seven additional samples were obtained from badgers trapped during TB control culling (five adults and two young).

### Rhodamine B Detection

Two hundred and thirteen hairs were positive for the presence of RhB, accounting for 33.0% of all the examined hair samples. The last positive sample was collected 177 days after the last day of bait deployment. Thirty eight samples (7.1%) gave inconclusive results, in that fluorescence was too low to ascertain positivity, they were thus excluded from the analysis. Because of the heterogeneity in hair growth (depending on the age and type of hair), loss of hair, and RhB persistence (which can last 177 days in our study and up to 24 weeks in 31), we were not able to discriminate whether badgers had consumed the baits in spring, summer, or both.

### Genetic Typing

Nine percent of hairs collected out of the total sampled (61/646) were discarded due to absence of bulb or bad quality of the sample. An amplification success rate of 67.8% was obtained for the 585 remaining samples. Reliable genetic profiles for identification were returned for 374 (64%) samples as 54 were contaminated, 142 had less than eight amplified microsatellites, and 15 could not be correctly assigned to one individual. Ninety-five consensus genotypes for 34 males and 61 females were identified from the 374 hair samples. Using at least eight microsatellites, we obtained a very low probability of identifying siblings [Prod (PI_sib_) estimation = 2.054 10^−8^], indicating a high confidence for individual identification (39).

The number of hair samples collected per individual ranged from 1 to 15 (mean = 3.9 ± 3.2) (see Supplementary Material 3). Among the 95 individuals, 28 (29.5%) were hair trapped only at the setts, 19 (20%) only at the pastures, and 42 (44.2%) both at setts and pastures. The seven trapped badgers provided genotypes that had been identified on hair traps either on setts or pastures or both.

Thirty-six individuals (37.9%) were identified after the first 2 weeks of hair collection and 70.5% were identified before the summer deployment.

The number of individuals identified per social group ranged from 0 to 14 with an average of 5.5 ± 3.7. The number of badgers identified per pasture ranged from 0 to 13 (mean = 5.4 ± 3.8).

### Bait Uptake

The number of baits available per badger, taking into account the total of the baits deployed (i.e., in spring and summer) ranged between 10 and 84 with an average of 28.2 ± 19.7 (Table 2). In summer, we did not observe any effect of the packaging on bait consumption. We excluded from the analysis the individuals having a single sample with inconclusive RhB detection result (*n* = 4). Nine individuals sampled at pasture hair traps were negative for RhB. As they could not be assigned to a baited sett, we chose to exclude them from the analyses at the individual and social group levels. As a result, RhB analyses were performed on 356 samples from 82 individuals. Among the samples that could not lead to individual identification, we found a similar proportion of negative/positive/inconclusive RhB samples than among the 374 hair samples allowing a proper identification.

#### Individual Level

At an individual level, 47.5% of the individuals had inconsistent results among their samples (see Supplementary Material 3). Forty-three badgers (52.4%) had RhB staining in at least one of their hair sample and were considered RhB positive. Thirty (70%) of these positive badgers had consumed the baits during the spring deployment (i.e., they were found positive before the summer deployment).

The number of samples collected was the only significant factor explaining the status of a badger: the higher the number of samples for a badger, the more likely it was to be positive for RhB (Table 3). The social group (i.e., random effect) explained 9% of the variability.

**Table 3:** Average model coefficients and *p*-value of the Wald test calculated for variables included in top models (ΔAICc < 4) explaining variation in bait uptake (detection of RhB in individual's hairs) by captured badgers (*n* = 82).

**Variables**	**Estimate**	**Lower**	**Upper**	***p*-value**
		**95% CI**	**95% CI**	**Wald test**
Intercept	−0.45	−1.86	1.08	0.54
Seasons (Spring+Summer)	0.99	−0.13	2.87	0.26
Samples^*^	**0.98**	**0.35**	**1.68**	**<0.01**
Zone (Zone B)	−0.20	−2.08	0.66	0.67
Caught at outlier sett (Yes)	−0.14	−1.90	0.77	0.734
Sex (Male)	−0.04	−1.31	0.82	0.86
Group size^*^	−0.03	−0.96	0.64	0.86

*Reference modalities are in bracket*.

* *Variables scaled. Significant result is in bold*.

#### Social Group Level

One social group (ID18, see Table 2) did not have any hair collected during the whole study (despite signs of activity observed on the ground and by video surveillance before and between the deployments) and was therefore excluded from this level of analysis. Twelve of the 14 remaining setts had at least one RhB-positive badger. The number of positive badgers per social group ranged between 0 and 8 with an average of 3.1, accounting for a mean proportion of 48.9% positive individuals per social group.

The first two axis of the FAMD explained 63.4% of the variability of the variables included in the dataset. The variables that most contributed to the definition of dimension one were *nb_baits* (17.3%), *cubs* (14.2%) *group_size* (12.9%), *zone* (12.4%), *seasons* (11.5%), and *prev_trapping* (10.5%). The variables that most contributed to the definition of dimension two were *seasons* (20.0%), *outlier* (18.8%), *zone* (15.5%), *prev_trapping* (15.2%), and *prop_pos_sett* (11.3%). *prop_pos_sett* mainly contributed to the third axis (15.2%) as well as *trapping* (37.7%), *outlier* (19.1%), and *group_size* (12.3%).

**Figure 5:**
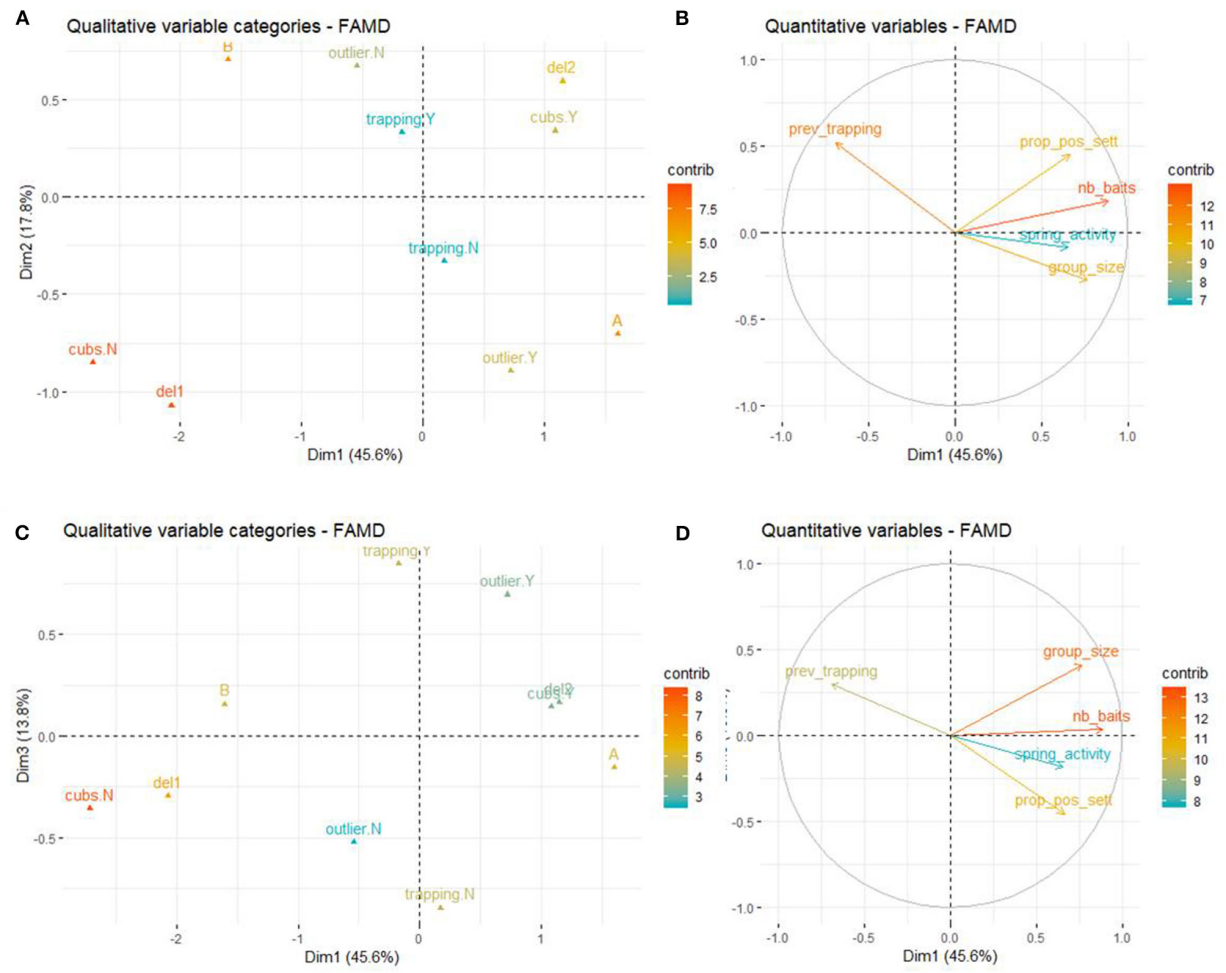
Results of the FAMD at the social group level. **(A)** Graph of the qualitative variables plotted on dimensions 1 and 2. **(B)** Graph of the quantitative variables plotted on dimensions 1 and 2. **(C)** Graph of the qualitative variables plotted on dimensions 1 and 3. **(D)** Graph of the quantitative variables plotted on dimensions 1 and 3.

A higher proportion of positive badgers by social group was associated with deploying baits in spring and summer compared to only spring, the absence of outlier sett, the absence of trapping during the deployment and zone B (compared to zone A). To a lesser extent, the presence of cubs and a higher number of baits were also associated with a higher proportion of positive badgers by social group (Figure 5).

The results of the univariate analysis showed that the proportion of positive badgers per social group was significantly higher in social group where cubs were present (Wilcoxon test, W = 4.5, *p* = 0.03) and where baits were delivered in spring and summer compared to just in spring (Wilcoxon test, W = 5, *p* = 0.02).

#### Pasture Level

Of the 13 pastures in the study, one was excluded from the analyses as no hair samples were collected. We found RhB-positive badgers on 10 out of the 12 pastures. The number of positive badgers per pasture ranged from 0 to 8 with an average of 2.75, accounting for a mean proportion of 50.6% positive badgers per pasture. As previously mentioned, nine individuals were hair trapped at five of the pastures traps (zero to five different badgers per pasture) but neither captured at any of the baited setts nor RhB positive. We included them to count the total number of badgers identified per pasture (variable *Nb_badgers*, see Table 1).

**Figure 6:**
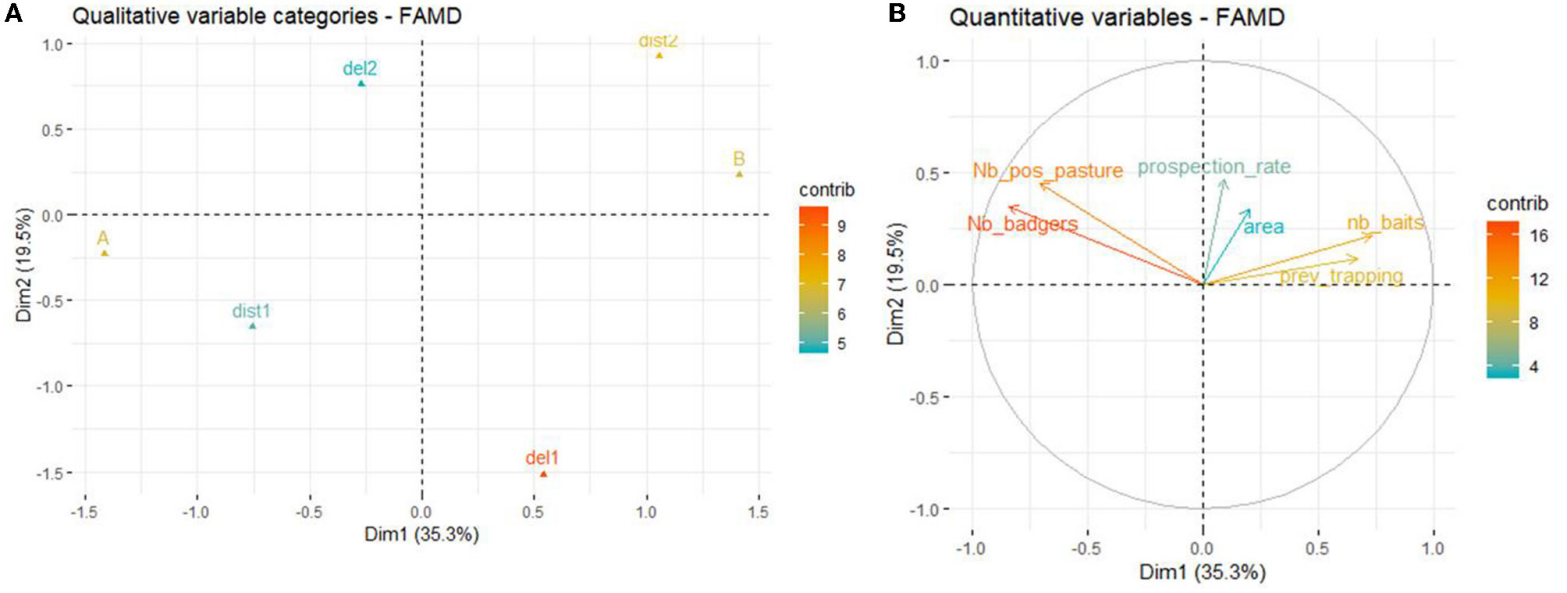
Results of the FAMD at the pasture level. **(A)** Graph of the qualitative variables plotted on dimensions 1 and 2. **(B)** Graph of the quantitative variables plotted on dimensions 1 and 2.

The first two axis of the FAMD explained 54.8% of the variability of the variables included in the dataset. The variables that most contributed to the definition of dimension one were *Nb_badgers* (22.3%), *zone* (19.9%), *nb_baits* (17.0%), *Nb_pos_pasture* (15.7%), and *prev_trapping* (14.1%). The variables that most contributed to the definition of dimension two were *seasons* (37.3%), *Dist_sett.pasture* (19.7%), *prospection_rate* (12.5%), and *Nb_pos_pasture* (11.8%) (Figure 6).

According to the FAMD, the number of positive badgers per pasture was positively associated with the total number of badgers identified at the pasture and zone A (relative to B) and negatively correlated with the number of baits deployed per badger as well as the intensity of previous trapping. A positive effect of deploying the baits in spring and summer (vs. only in spring) as well as a higher prospection rate around the pasture were also found. The number of positive badgers was not associated with a baited setts located within the pasture vs. outside the pasture.

The univariate analysis provided one single significant result: The number of positive badgers per pasture was significantly associated with the total number of badgers identified at the pasture (Pearson correlation test, cor = 0.73, *p* = 0.006).

## Discussion

Using oral vaccination of wildlife could be an effective and ethical tool to manage TB at the wildlife-livestock interface. However, the development of such a vaccine is very challenging as it requires an efficient and safe vaccine and a deployment strategy leading to a sufficient vaccination coverage. Baits delivery systems and vaccine models aimed at reducing TB prevalence and transmission have been tested in different wild species around the globe (2, 7, 42, 43). Here, using a non-invasive method combining biomarker detection and genotyping on badger hairs, collected at setts and nearby pastures, we estimated that approximately 50% of badgers have consumed one or more of the baits for each level of the study (individual badger, the social groups, and at pastures levels). These results show encouraging levels of bait uptake. Moreover, we identified favorable and limiting factors that could influence bait uptake, which should be taken into account in the optimization of future vaccine deployments.

### Advantages and Limits of the Method Used

The results of the biomarker detection confirmed that RhB can persist for a long time in hair after consumption (last positive sample collected 177 days after the bait deployment), which is concordant with a previous study [24 weeks = 168 days, also in badger hair (31)]. However, the sensitivity of the method may depend on the number of hairs collected per sample and per individual as shown by the results of the GLMM at the individual level. This could also be the case if this method is used in other species. Here, the molting cycle and the age of the badgers may explain false negative results (31, 32). In badgers, molting starts in July and ends in January with a progression of the hair loss and growth from the front to the rear and from the dorsal region toward the abdomen (44). This pattern is different in cubs (continuous hair growth) and juveniles of 1–2 years old (molting starts 1 or 2 months before adults) (34). As a result, false negatives may have occurred in our study during the molting period, i.e., from July to October or even earlier in juveniles. This lack of sensitivity could have resulted in an underestimation of the bait uptake. In addition, this biomarking method did not allow discriminating whether badgers had eaten the baits in spring, summer, or both because of the heterogeneous growth of the hair and RhB persistence.

Another biomarker, the propyl ionphenoxic acid (IPA) has also been used to determine the level of bait uptake by badger. IPA is less persistent than RhB (16 weeks vs. 24 weeks) and the false negative may also occur depending on the chosen threshold value (26). Moreover, using IPA requires invasive operations such as trapping, anesthetising, and blood sampling. Results could also be skewed by trap wariness by some badgers in the studied population (26, 45). Here, badgers may have avoided passing under the hair traps (as suggested by the absence of hair sample collected at one sett), but this method is thought to sample a greater proportion of the population than trapping, which is required when using alternative biomarkers such as IPA (26, 36). In addition, avoidance of the hair traps was reduced by ensuring that they were installed a few weeks before sampling, to allow a period of habituation and by using pastures' fences already present in the badgers' environment.

Regarding the genotyping analysis, we obtained a very low probability of identifying siblings allowing discarding reasonably double counting of one individual. Although it is likely that not all the individuals in the population were detected, due to the sampling limitations or due to shortcomings in genotyping (i.e., contamination or amplification failures), the number of “missed individuals” may have been limited by the duration of the study (6 months) and the spatial coverage of the hair trapping. For example, Scheppers et al. (36) identified all the members (confirmed by capture–marking–recapture method) of nine groups after 4 weeks of hair collection. Moreover, except on one sett where no hair sample was collected, genetic typing provided equal or higher number of badgers per social group than what was recorded by video-surveillance, giving confidence in the number of individuals found by the genetic tool. In addition, as we found a similar proportion of negative/positive/inconclusive RhB samples between the ones that lead to a genetic identification and the ones that could not provide a reliable genetic profile, we can reasonably assume that genotyping errors did not influence the final results of bait uptake.

### Bait Uptake by Badgers

The level of bait uptake found was lower than what was reported in British studies using similar baits (or similar baits components) but different biomarking method. For instance, more than 90% of the badgers consumed the baits in two studies carried out in England (25, 46). An aversion to the baits due to the presence of RhB has been observed in other species like the white-tailed deer (43) but, as acknowledged in Section Bait Preparation and Deployment, no loss of palatability was observed for the badger when RhB was integrated to the bait's components. The use of other biomarkers in the British studies is not believed to explain the higher bait uptake they observed. In these study sites, the badger population had been baited for a long time for scientific purpose with baits containing some of the same components as the ones used in PT and were thus less neophobic. A bait uptake of 83% was reported by Carter et al. (26) in a naïve badger population in southwest England. In this case, differences in badgers' behavioral response to bait delivery, in natural resource availability and a higher quantity of baits deployed per sett (165 bait portions per main sett), could explain the higher uptake, which, however, varied considerably between groups. Furthermore, another study (not reported here) carried out at the same time on the same setts, using video surveillance, showed a significant impact of non-target species on baits removal, especially by red foxes and birds (observed on 13 out of the 15 setts with some consumption of the baits detected), even when baits were delivered inside the holes. Cattle might also be attracted by the bait if they have access to it (47). Competition for baits between badgers and non-target species could thus have affected bait uptake by badgers to a bigger extent than in the UK (22, 25, 26, 35). Like in the present study, no sex effect was observed on the bait uptake assessed in the UK (25, 26, 46). The age of the badgers could not be estimated at the individual level, but the presence of cubs (attested by video surveillance) was one of the drivers associated with higher proportion of positive badgers per social group. According to video surveillance data collected on the studied setts, cubs emerged in April and most likely gained access to the baits during the spring deployment. Moreover, young may be less wary than adults toward the baits. This is consistent with British studies where no age effect was found at the individual level, suggesting no competitive advantage of the adults upon the young (25).

Although the model selected to explain RhB result at the individual level did not highlight any effect of the zone, social groups from zone A tended to be bigger, with a higher number of cubs and more active than groups from zone B (especially during the spring deployment) (see Table 2 and Figure 4). As suggested by the FAMD results at the social group and pasture levels, this contrast might be linked to the difference in the trapping intensity implemented in the last 9 years between area A and B (see Supplementary Material 1). Intense trapping in zone B resulted in reduced proportion of occupied main setts (28). It is also possible that a behavioral component occurred in area B: By being subjected to a more intense trapping activity, badgers from zone B may have been more sensitive to human disturbance than badgers from zone A. As a consequence, human activities associated with setts monitoring, baits delivery, and hair collection may have scared away the badgers from zone B more than in zone A. In addition, according to the FAMD result at the social group level, ongoing trapping activity was negatively associated with bait uptake, suggesting here again that trapping is detrimental to an optimal bait uptake. However, no effect of the zone or of the badgers' spring activity was found on the individual and, at the social group level, a higher bait uptake was surprisingly associated with zone B rather than zone A (FAMD). We therefore hypothesize that badgers from zone B were indeed fewer than in zone A and moved away from their sett during the spring deployment but came back (as shown by the activity that increased between the deployment, see Figure 4) and consumed baits sometimes after the deployment. An experimental study showed a notable and more rapid decrease of CFU BCG per bait after ~7 days of storage at 4 and 20°C (23). In the perspective of a real deployment using the BCG, this lag that we observed between the bait deployment and consumption by badgers might jeopardize the efficacy of the vaccine. This highlights that one of the big challenge in the deployment of an oral TB vaccine using BCG, whatever the targeted species, is to ensure that the BCG remains viable and thus keeps its effectiveness when the target species consume the bait. It is also noteworthy that the badgers' activity was higher in zone A than zone B during the spring deployment but did not differ in summer, groups being less active in both zones at this season. This observation might reflect inconstant occupations of the main sett, and a higher time spent in outlier setts usually occurring during summer (29, 48), regardless of human disturbance.

Feeding an active detected outlier sett was not associated with higher bait uptake at the group level. As in the UK (26), we could have expected a better bait uptake when outlier setts were fed vs. not fed. Only 19 out of the 82 individuals were hair trapped at a baited outlier sett. Among these 19, none was trapped during the bait deployment and only two were trapped before the sixth day following the first day of baits deployment. The use of outlier setts by the badgers of the studied population might have not matched the exact period of baits deployment. Alternatively, baits delivery could have triggered a similar behavior as at the main sett, leading the badgers to move to an undisturbed sett. In addition, inconstant residency at outlier sett by badgers could have supported the use of the area by non-target species.

The biomarking method did not allow to discriminate whether badgers had eaten the baits in spring, summer, or both. We implemented different protocols design in spring and summer and were therefore not able to compare the bait uptake between the two seasons. We found at the social group and pasture levels that the bait uptake was better for setts that were fed in both seasons compared to just in spring. In the five groups that were not fed in summer, we did not observe any cubs in four of these five groups. This unbalanced sample design could have biased the analysis in the sense that the setts fed only in spring corresponded to groups having a less favorable characteristic for bait uptake. It is also noteworthy that 70% of the RhB-positive individuals were positive before the summer deployment, indicating that a majority of the badgers that consumed baits in summer had already eaten some in spring. Spring is associated with less food availability and the emergence of cubs, which are both favorable factors for a higher bait uptake. Moreover, despite having implemented a less disturbing protocol in summer, we observed a general decrease in badger activity at this season most likely linked to seasonal movement (29) and thus not favorable to bait consumption. However, we did not observe on the ground any effect of the baits' packaging tested only in summer, confirming that this packaging does not seem to be detrimental to bait consumption. Despite what our results seemed to show, the benefit of targeting more badgers in summer following a spring deployment is questionable and should be considered from a cost-benefit point of view. This bait consumption pattern related to seasons might be different depending on the context and the targeted species.

The group size was not well-correlated with the number of baits available per individual. This result reflects that our sett scoring based on the number of active holes was not always well-related to the real number of resident badgers. However, we did not find any effect of the group size or the number of baits deployed per individual on the bait uptake, suggesting a sufficient number of baits deployed allowing each resident badger to consume at least one bait (and thus, one dose of vaccine if present). In the UK, Carter et al. (26) deployed a fixed number of baits per social group and detected an effect of the group size consistent with competition for baits. Our bespoke strategy seems to be more cost-effective despite the difficulty to assess accurately the size of the group based on field signs (49).

A high proportion (83%, 45 out of 54) of the badgers identified on the pastures was assigned to a baited sett. These results indicate that the population of badgers that were baited at the setts also visited the pastures. Regarding the 9 individuals that could not be ascribed to a known social group, we cannot rule out that we missed them at the baited setts either because they were not hair trapped or because the genotyping failed to identify them. However, as previously discussed, we are reasonably confident in having identified most of the members of the studied social groups. Alternatively, these badgers could come from other setts not detected when prospecting around the pastures. It is noteworthy that these 9 badgers were not evenly distributed among the pastures with five being found on the same pasture where we were not able to survey densely vegetated areas nearby. In that context, it is possible that we missed setts, located in these areas. Finally, we cannot exclude the possibility of badgers coming from setts located further away than the 300-m radius that we prospected, as distances made by badgers around their sett on several months exceed 300 m (29, 50).

This study provides a first insight into a potential vaccination coverage by using the candidate bait developed by the APHA, in the context of TB control measured applied in France. A further step would be to assess if such a level of bait uptake would significantly reduce badgers *M. bovis* excretion and transmission from and between badgers. Several models had simulated the effects of vaccination in badgers on prevalence reduction in badgers and cattle. In the Republic of Ireland, a model based on a vaccination field trial, using BCG administrated directly in oral mucosa, set with prevalence in badgers of 18% and a vaccine efficacy of 59%, predicted that a vaccine coverage exceeding 30% would make eradication of *M. bovis* in badgers in Ireland feasible, provided that the current control measures also remain in place (51). A field trial carried out in the UK (badger density = 25 badgers/km^2^ and TB prevalence = 35%−53%) showed that intramuscular injection of BCG reduced by 76% the risk of free-living vaccinated individuals testing positive to a diagnostic test combination to detect progressive infection. Furthermore, when more than a third of their social group had been vaccinated, the risk to unvaccinated cubs was reduced by 79% (18). The bait uptake of roughly 50% found in our study at the individual, social group, and pasture levels is therefore promising for the purpose of controlling TB in badgers in France, if assuming the assumptions of models and trials applied to the British Isles situation are also applicable to France. However, TB prevalence and badgers' density are much higher in the UK and Ireland, and a detrimental perturbation effect of badger culling on cattle incidence (in the UK at least) has been observed (12). These models are sensitive to badgers' density and TB prevalence (14, 15), and different outcomes are likely to arise when applied to the French situation. In addition, the effect of BCG administration through baits might not be strictly equivalent to direct delivery to oral mucosa or intramuscular injection. Modeling the effect of vaccination in the French situation taking into account the importance of external sources (i.e., cattle but also other wild hosts such as wild boars and deer) and using this bait uptake as a vaccine coverage parameter would be necessary to explore more deeply the benefit of oral vaccination. The duration and the frequency of the vaccination campaigns and the area to be covered are other pending questions. The protocol that we used in our study mimicked a “reactive vaccination” as we selected the setts to be baited in the close vicinity of pastures that should be protected against the visit by infected badgers. The result of 50.6%, in average, of badgers that had consumed the baits at the pasture level could also be used to evaluate the value of such a strategy, providing that it would be more cost-effective than deploying a widespread vaccination.

## Conclusion

The present study demonstrated that a bait uptake of 52.4% was achieved by deploying an average of 28 baits per badger, using the candidate bait recently developed by APHA, down setts, once in spring and once in summer. The study highlighted the positive influence of cubs' presence and the negative effect of previous and ongoing trapping on bait uptake. Deploying the baits when cubs are weaned and emerge (April to May) appeared to be a good strategy to increase the bait uptake, especially by cubs. The benefit of deploying baits in summer and at the outlier setts is less clear as we observed an inconstant occupancy of the main setts in summer and did not find any positive effect of baits delivery at outlier setts. As a consequence, we could propose to first deploying the baits in early spring to target the adults as cubs are not yet weaned, natural food is still scarce, and adults need to replenish. A second deployment could be made in May to June, when cubs have emerged and when the main sett is still permanently occupied. Tailoring the number of baits to be deployed by coarsely classifying the sett based on field signs seemed valuable to increase the chance for all members of the group to gain access to the baits. The competition with non-target species, various and abundant in this area, should also be taken into account. Consumption of the baits by cattle should be avoided as it may interfere with TB diagnostic tests. The group where 100% of the members had consumed the baits received 20 baits per badger. This threshold could be used as a base for further deployment, providing that data on badgers' density are available. It would require a high number of baits to be deployed and thus an affordable cost for the vaccine. Strategies aiming to cope with the behavioral response of the badger population previously intensively trapped should be addressed, as this behavior occurred in a population where vaccination would be the most relevant to implement. Moreover, the study showed that a “reactive baiting” in a radius of 300 m around targeted pastures could be a cost-effective strategy to select the setts to be baited and protect cattle from badgers contamination. In the present experimental design, the pastures were limited in number and scattered, but a more widespread deployment could be expected in a real vaccination program where a whole infected area, with contiguous pastures, would be targeted. Such a strategy might optimize the bait uptake by reaching the disturbed and “super rangers” badgers (52). Further research is therefore needed to complete and confirm these results, as well as research assessing vaccine efficacy through this candidate bait. Models are also necessary to estimate if the level of vaccine coverage estimated here would be sufficient to control TB in the French multi-hosts system.

## Data Availability Statement

The original contributions presented in the study are included in the article/Supplementary Material, further inquiries can be directed to the corresponding author/s.

## Ethics Statement

Ethical review and approval was not required for the animal study because no animal was manipulated or extracted from its natural environment and we used non-invasive methods (camera-traps and hair trapping) to collect the data.

## Author Contributions

SRo and SRu conceptualized the study. AP, SRo, SL, CR, and SRu designed the methodology. AP and JD carried out the field work and collected and processed the data (hair samples and video footages). CR and AP analyzed the hair samples for RhB detection. MJ performed the genetic analysis. AP and SRu performed the statistical analysis. SM and SL were in charge of the bait providing and preparation. AP wrote the manuscript. All authors helped in the drafting or reviewed the manuscript critically. All authors contributed to the article and approved the submitted version.

## Funding

This study was financed by the French Ministry for Agriculture and Food (MAA) (Agreement No. 2017-397), the National Groupement de Défense Sanitaire (GDS France), the local Groupement de Défense Sanitaire (GDS 21), and the Wildlife and Hunting Agency (ONCFS now being called French Office for Biodiversity – OFB). This work was also funded by the bTB research budget (Project SE3247) held and administered centrally by Defra on behalf of England, Scotland, and Wales.

## Dedication

The authors wish to dedicate this work to the beloved memory of Sophie Rossi, who sadly passed away during the preparation of this manuscript. She dedicated her career to further the understanding of wildlife diseases and improve wildlife health, and her passionate work will remain an inspiration to us all.

## Conflict of Interest

The authors declare that the research was conducted in the absence of any commercial or financial relationships that could be construed as a potential conflict of interest.

## Publisher's Note

All claims expressed in this article are solely those of the authors and do not necessarily represent those of their affiliated organizations, or those of the publisher, the editors and the reviewers. Any product that may be evaluated in this article, or claim that may be made by its manufacturer, is not guaranteed or endorsed by the publisher.
