# Supplementary material for: Estimation of Bait Uptake by Badgers, Using Non-invasive Methods, in the Perspective of Oral Vaccination Against Bovine Tuberculosis in a French Infected Area
Source: Front Vet Sci. 2022 Mar 9;9:787932. doi: 10.3389/fvets.2022.787932 (PMC8961513; doi:10.3389/fvets.2022.787932)
Supplement: Supplementary file 1 [file Data_Sheet_1.pdf]

SUPPLEMENTARY DATA 1

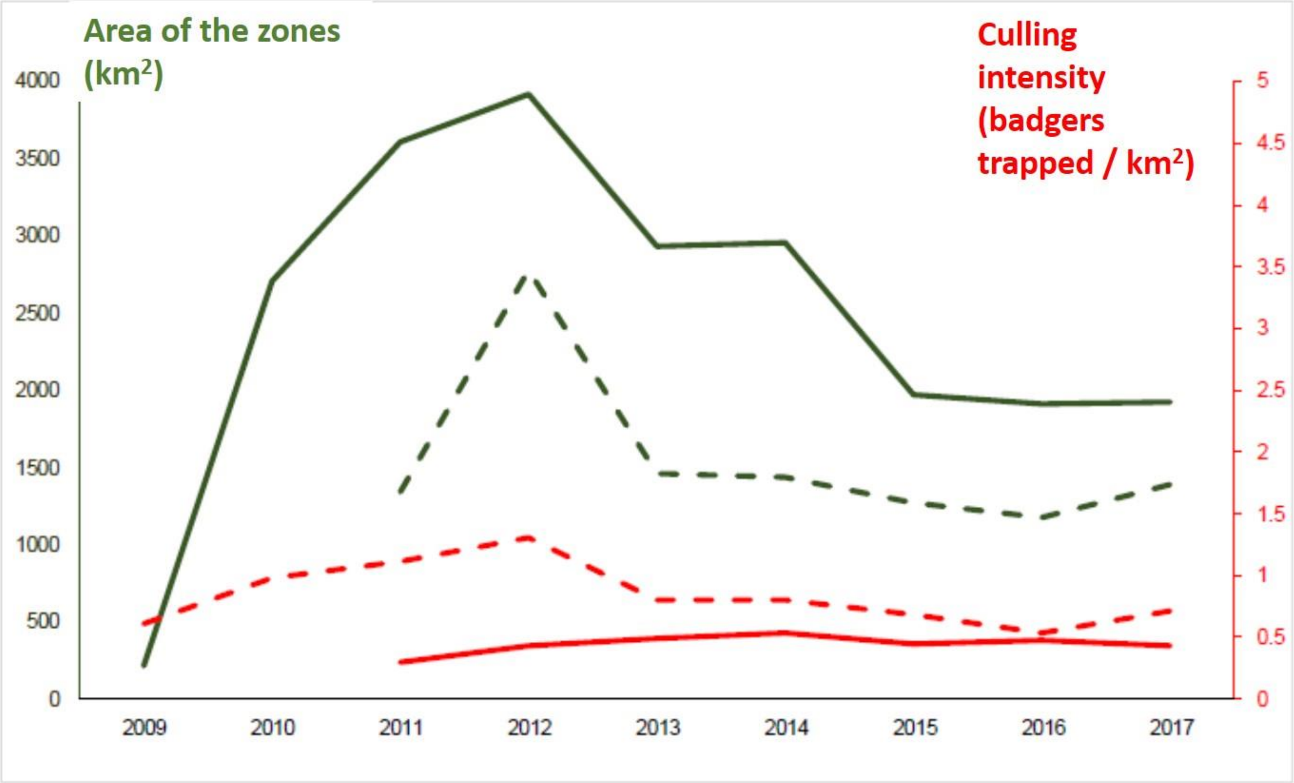

Figure 1 : Evolution of the size of the area (solid lines) where badgers culling was implemented and culling intensity (dotted lines). Red lines correspond to the buffer zone (including area A) and green lines correspond to the infected area (including area B) (Source : ANSES, 2019, see 27 in the manuscript's references).
