# Supplementary material for: Estimation of Bait Uptake by Badgers, Using Non-invasive Methods, in the Perspective of Oral Vaccination Against Bovine Tuberculosis in a French Infected Area
Source: Front Vet Sci. 2022 Mar 9;9:787932. doi: 10.3389/fvets.2022.787932 (PMC8961513; doi:10.3389/fvets.2022.787932)
Supplement: Supplementary file 2 [file Data_Sheet_2.pdf]

**SUPPLEMENTARY MATERIAL 2**

| Mix | Microsatellite locus | Size (bp) | Fluorescent dye | Final concentration (μM) | Reference |
|---|---|---|---|---|---|
| A | 02-Mel5 | 91-127 | FAM | 0.20 | Bijlsma *et al.* 2000 |
| A | 04-Mel14 | 171-195 | NED | 0.20 | Huck *et al.* 2008 |
| A | 08-Mel105-V2 | 136-175 | VIC | 0.20 | Carpenter *et al.* 2003 |
| A | 09-Mel106 | 191-226 | VIC | 0.20 | Carpenter *et al.* 2003 |
| A | 10-Mel109 | 101-132 | PET | 0.20 | Carpenter *et al.* 2003 |
| A | 13-Mel114 | 199-261 | PET | 0.20 | Carpenter *et al.* 2003 |
| A | 15-Mel116 | 108-135 | NED | 0.20 | Carpenter *et al.* 2003 |
| A | 17-Mel125b | 148-171 | FAM | 0.20 | Huck *et al.* 2008 |
| A | 18-Mel128-V2 | 218-242 | FAM | 0.20 | Huck *et al.* 2008 |
| A | 21-Mel499 | 339-363 | FAM | 0.20 | Annavi *et al.* 2011 |
| A | 24-Mel558 | 237-255 | VIC | 0.20 | Annavi *et al.* 2011 |
| A | 26-Mel-SRYa-V2 | 150 | NED | 0.20 | Huck *et al.* 2008 |
| B | 01-Mel1 | 259-290 | FAM | 0.20 | Huck *et al.* 2008 |
| B | 03-Mel7 | 140-167 | NED | 0.20 | Huck *et al.* 2008 |
| B | 05-Mel101-V2 | 126-166 | PET | 0.20 | Carpenter *et al.* 2003 |
| B | 06-Mel102 | 181-205 | VIC | 0.20 | Carpenter *et al.* 2003 |
| B | 07-Mel104 | 302-331 | VIC | 0.20 | Carpenter *et al.* 2003 |
| B | 11-Mel112 | 384-432 | FAM | 0.20 | Carpenter *et al.* 2003 |
| B | 12-Mel113 | 107-134 | FAM | 0.20 | Carpenter *et al.* 2003 |
| B | 14-Mel115 | 319-351 | FAM | 0.20 | Carpenter *et al.* 2003 |
| B | 16-Mel117-V2 | 182-213 | PET | 0.20 | Carpenter *et al.* 2003 |
| B | 19-Mel129 | 191-241 | FAM | 0.20 | Carpenter *et al.* 2003 |
| B | 20-Mel451 | 196-233 | NED | 0.20 | Annavi *et al.* 2011 |
| B | 22-Mel522 | 225-285 | VIC | 0.20 | Annavi *et al.* 2011 |
| B | 23-Mel551-V2 | 234-264 | PET | 0.20 | Annavi *et al.* 2011 |
| B | 25-Mel-SRYa | 125 | NED | 0.20 | Huck *et al.* 2008 |

**REFERENCES**


Annavi, G. et al. 2011. Characterisation of twenty-one European badger (Meles meles) microsatellite loci facilitates the discrimination of second-order relatives. Conservation Genetics Resources 3:515–518.

Bijlsma, R., M. Van de Vliet, C. Pertoldi, R. C. Van Apeldoorn, and L. Van De Zande. 2000. Microsatellite primers from the Eurasian badger, Meles meles. Molecular Ecology 9:2215–2216.

Carpenter, P. J., D. A. Dawson, C. Greig, A. Parham, C. L. Cheeseman, and T. Burke. 2003. Isolation of 39 polymorphic microsatellite loci and the development of a fluorescently labelled marker set for the Eurasian badger (Meles meles) (Carnivora: Mustelidae). Molecular Ecology Notes 3:610–615.

Huck, M., A. C. Frantz, D. A. Dawson, T. Burke, and T. J. Roper. 2008. Low genetic variability, female-biased dispersal and high movement rates in an urban population of Eurasian badgers Meles meles. Journal of Animal Ecology 77:905–915.
