# Supplementary material for: Estimation of Bait Uptake by Badgers, Using Non-invasive Methods, in the Perspective of Oral Vaccination Against Bovine Tuberculosis in a French Infected Area
Source: Front Vet Sci. 2022 Mar 9;9:787932. doi: 10.3389/fvets.2022.787932 (PMC8961513; doi:10.3389/fvets.2022.787932)
Supplement: Supplementary file 3 [file Data_Sheet_3.docx]

**SUPPLEMENTARY DATA 3**


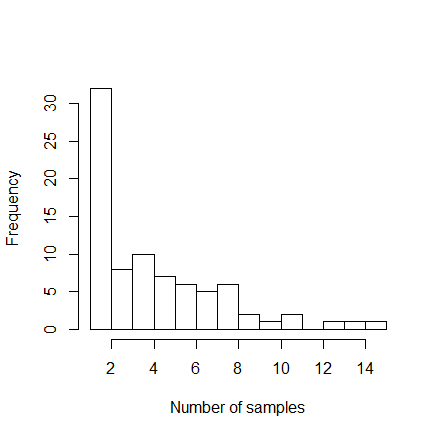
Figure 1 : frequency of the number of samples collected per badger


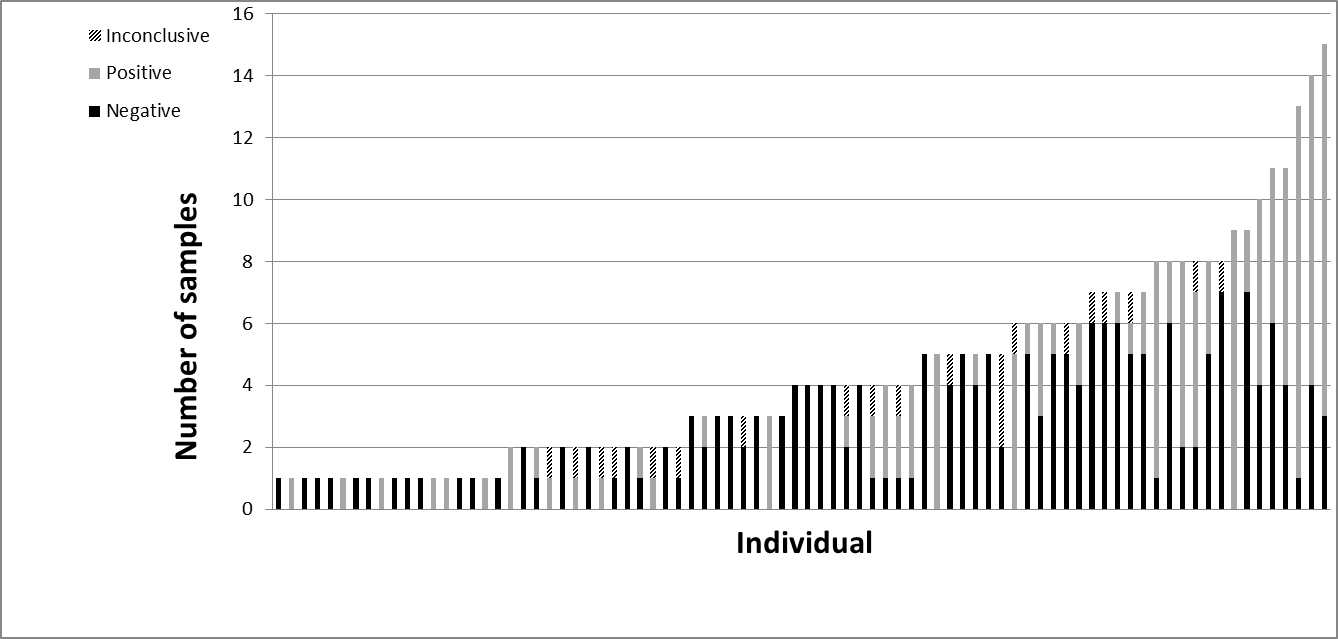


Figure 2 : distribution of the RhB results for the hair samples belonging to each badger (N= 95 individuals)


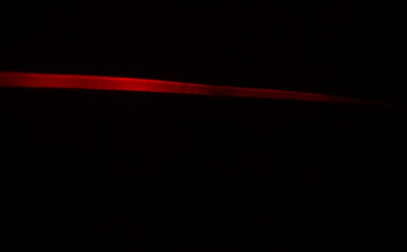

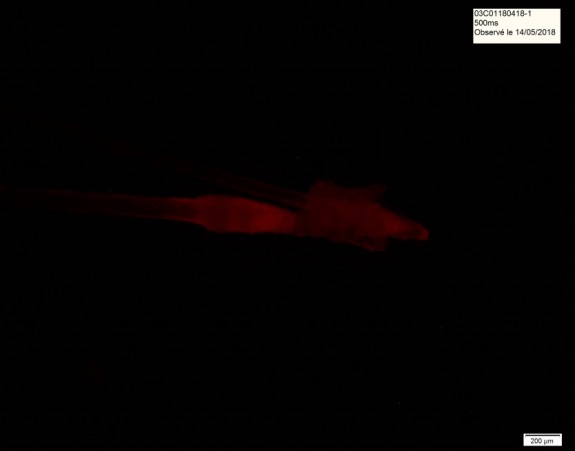


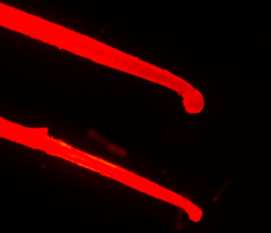


Bulbs, day 2 Bulb day 23 Apex, day 64


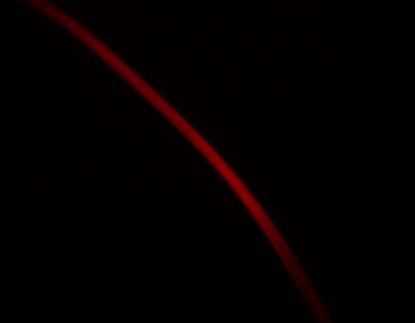


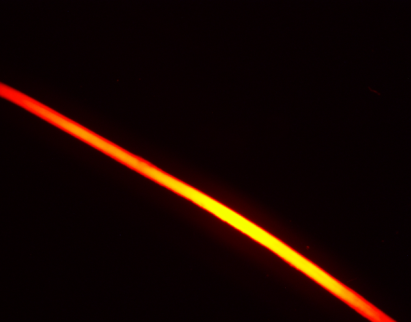


Apex, day 91 Half shaft, day 177

Figure 3 : examples of Rhodamine B fluorescence observed on hairs of badger at different delay of hair capture (the number of day is the delay between delivery of RhB baits and hair collection). Magnification x 4, Olympus fluorescence microscopy system
